# Supplementary figures and images for: Triaging and referring in adjacent general and emergency departments (the TRIAGE trial): A cluster randomised controlled trial
Source: PLoS One. 2021 Nov 3;16(11):e0258561. doi: 10.1371/journal.pone.0258561 (PMC8565772; doi:10.1371/journal.pone.0258561)

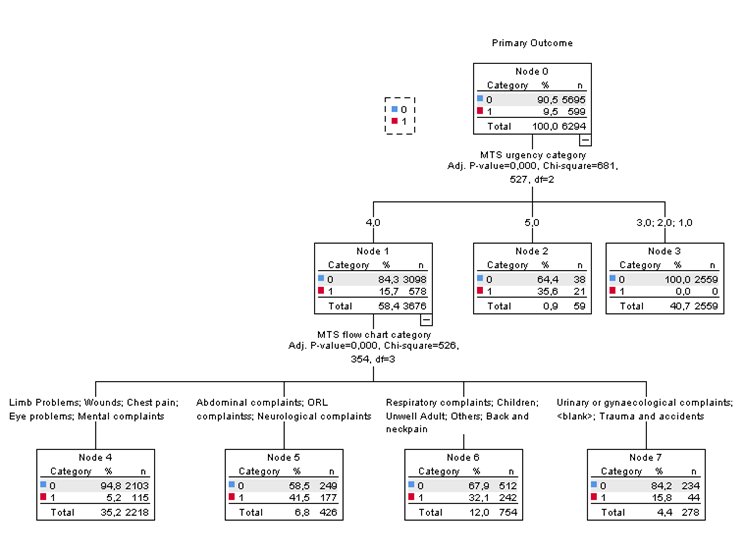

Supplement: S1 Fig — MTS: Manchester Triage System. ORL: Otorhinolaryngology. (PNG) [file pone.0258561.s002.png]

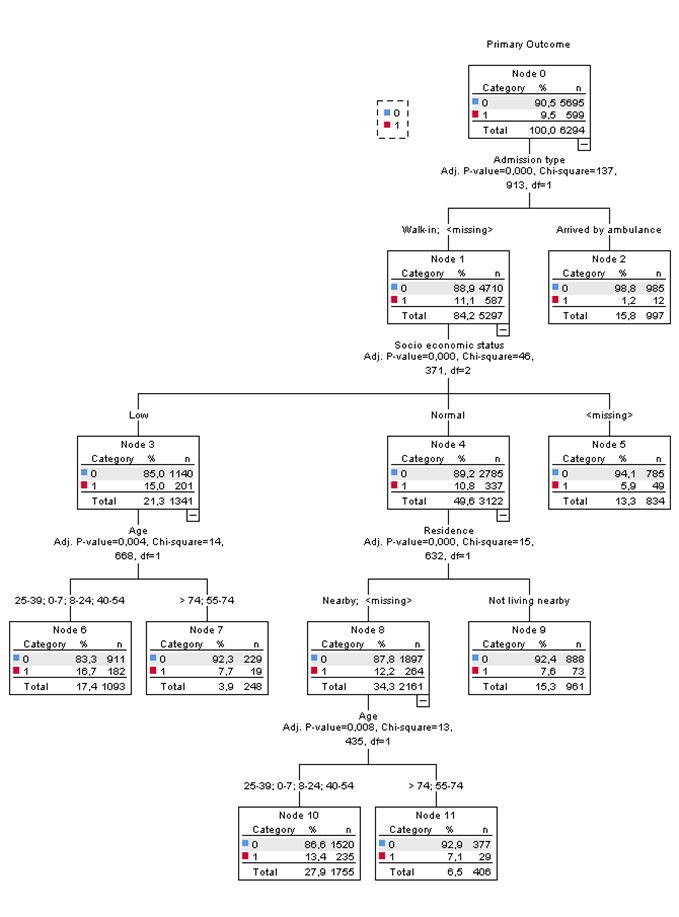

Supplement: S2 Fig — (PNG) [file pone.0258561.s003.png]

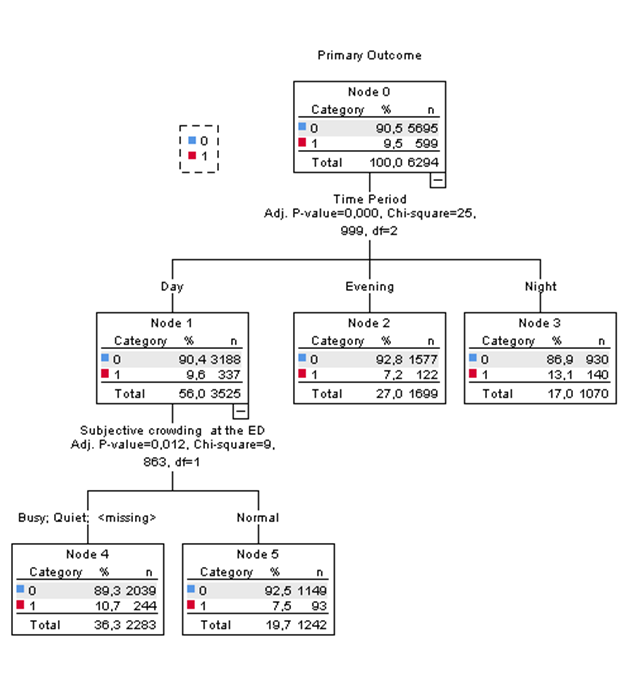

Supplement: S3 Fig — (PNG) [file pone.0258561.s004.png]

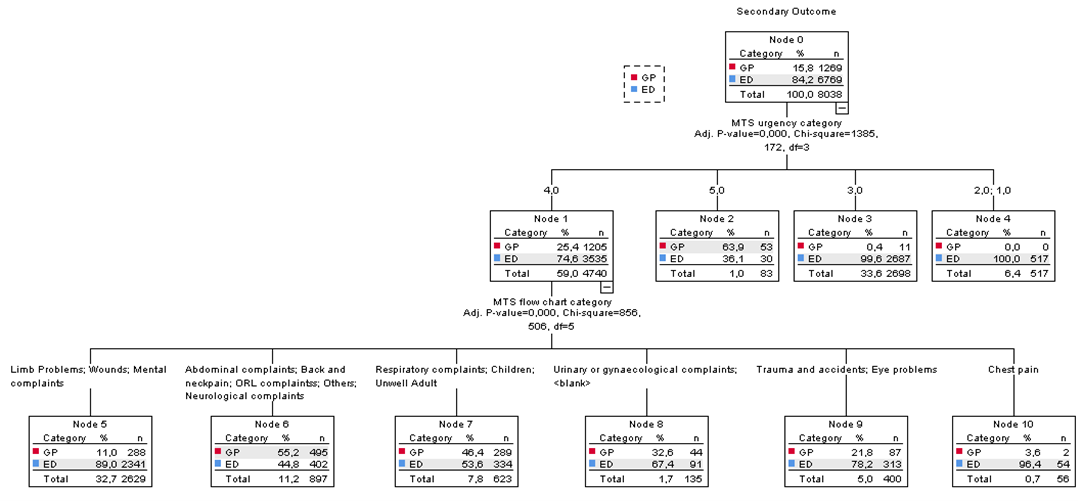

Supplement: S4 Fig — ED: Emergency Department. GP: General Practice. MTS: Manchester Triage System. ORL: Otorhinolaryngology. (PNG) [file pone.0258561.s005.png]

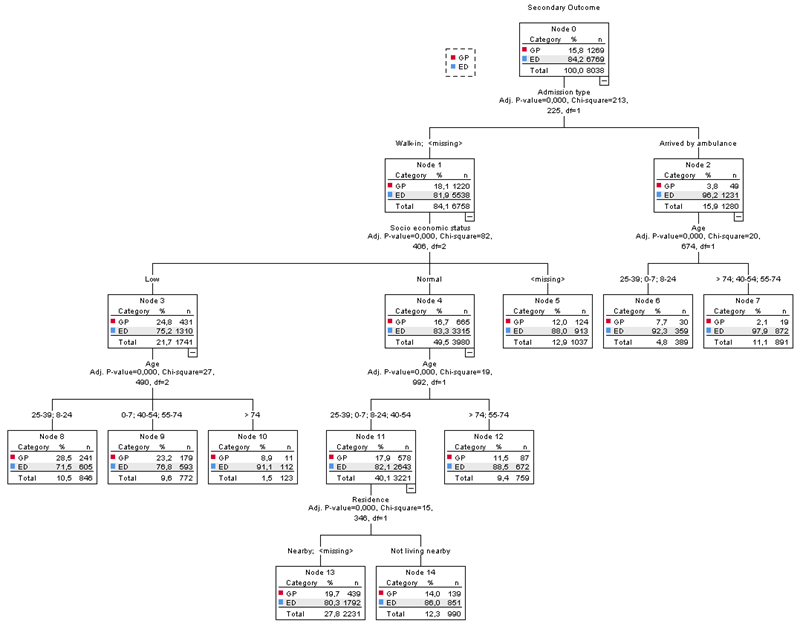

Supplement: S5 Fig — ED: Emergency Department. GP: General Practice. (PNG) [file pone.0258561.s006.png]

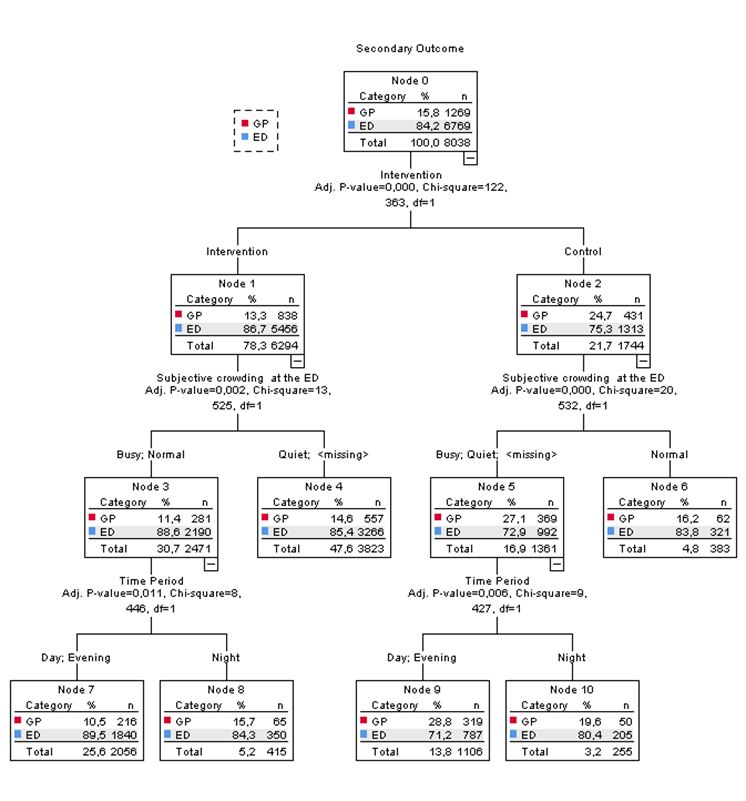

Supplement: S6 Fig — ED: Emergency Department. GP: General Practice. (PNG) [file pone.0258561.s007.png]

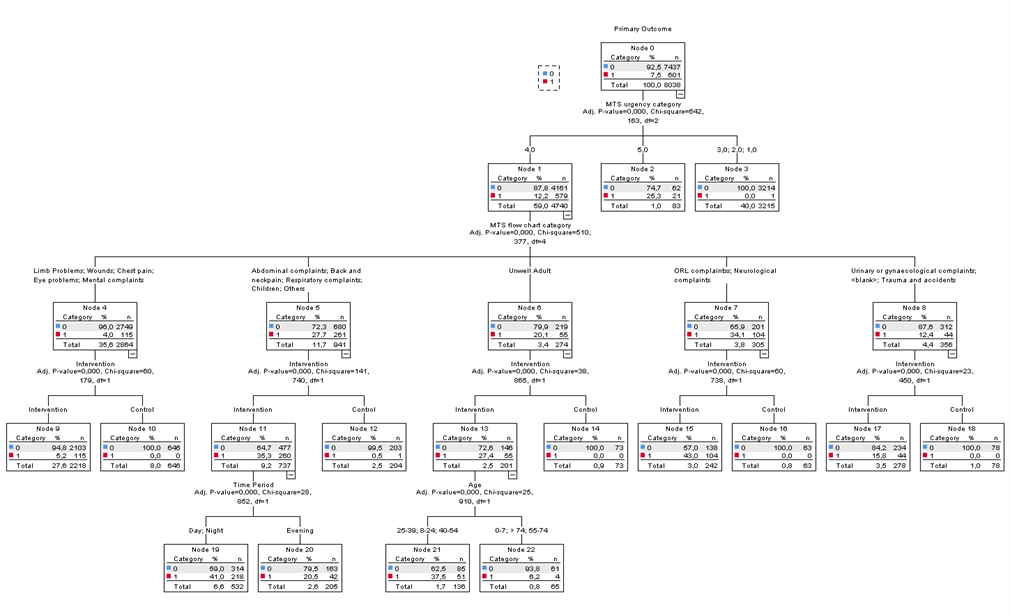

Supplement: S7 Fig — ED: Emergency Department. GP: General Practice. MTS: Manchester Triage System. ORL: Otorhinolaryngology. (PNG) [file pone.0258561.s008.png]
