# Supplementary material for: Triaging and referring in adjacent general and emergency departments (the TRIAGE trial): A cluster randomised controlled trial
Source: PLoS One. 2021 Nov 3;16(11):e0258561. doi: 10.1371/journal.pone.0258561 (PMC8565772; doi:10.1371/journal.pone.0258561)
Supplement: S1 Table — For categorical variables with more than four categories, the categories with the highest and lowest secondary outcome are reported. DF: degrees of freedom. MTS: Manchester Triage System. ED: Emergency Department. ORL: Otorhinolaryngology. *: Only for urgency categories four and five because the primary outcome was zero in the other categories. (DOCX) [file pone.0258561.s009.docx]

**S1 Table.** Bivariate analysis of the secondary outcome (all participants excluding those with a missing triage advice). For categorical variables with more than four categories, the categories with the highest and lowest primary outcome are reported.

| **Determinant** | **N** | | **Mean secondary outcome** | **DF** | **Category** | **Estimate** | **Wald Chi²** | **P-value** | | **Odds ratio (95%CI)** |
| --- | --- | --- | --- | --- | --- | --- | --- | --- | --- | --- |
| **Patient’s presentation** | | | | | | | | | | |
| MTS urgency category* | 4823 | | 26.1% | 1 | 4: Standard |  | | | | 1 |
|  |  |  |  |  | 5: Non-urgent | 1.65 | 50.8 | <0.01 | | 5.18 (3.30 to 8.15) |
| MTS flow chart category | 7978 | | 15.7% | 14 | Unwell adult |  | | | | 1 |
|  |  |  |  |  | ORL Complaints | 1.13 | 64.2 | <0.01 | | 3.08 (2.34 to 4.06) |
|  |  |  |  |  | Chest pain | -2.84 | 23.3 | <0.01 | | 0.06 (0.02 to 0.19) |
| **Patient characteristics** | | | | | | | | | | |
| Age | | 8038 | 15.8% | 5 | 0-7 years | 0.21 | 3.7 | **0.05** | | 1.23 (1.00 to 1.52) |
|  |  |  |  |  | 8-24 years | 0.25 | 6.6 | 0.01 | | 1.28 (1.06 to 1.55) |
|  |  |  |  |  | 25-39 years | 0.19 | **4.0** | 0.05 | | 1.21 (1.00 to 1.45) |
|  |  |  |  |  | 40-54 |  | | | | 1 |
|  |  |  |  |  | 55-74 | -0.39 | 11.1 | <0.01 | | 0.68 (0.54 to 0.85) |
|  |  |  |  |  | >74 | -1.02 | 41.6 | <0.01 | | 0.36 (0.27 to 0.49) |
| Admission type | 8034 | | 15.8% | 1 | Walk-in |  | | | | 1 |
|  |  |  |  |  | Arrived by ambulance | -1.71 | 131.5 | | <0.01 | 0.18 (0.14 to 0.24) |
| Sex | 8038 | | 15.8% | 1 | Female |  | | | | 1 |
|  |  |  |  |  | Male | -0.08 | 1.61 | 0.20 | | 0.93 (0.82 to 1.04) |
| Residence | 8012 | | 15.8% | 1 | Nearby |  |  | | | 1 |
|  |  |  |  |  | Not living nearby | -0.28 | 16.8 | <0.01 | | 0.75 (0.65 to 0.86) |
| Socioeconomic status | 6788 | | 16.7% | 1 | Normal |  | | | | 1 |
|  |  |  |  |  | Low | 0.45 | 45.1 | <0.01 | | 1.57 (1.38 to 1.79) |
| **Timing of presentation** | | | | | | | | | | |
| Intervention | 8038 | | 15.8% | 1 | Intervention |  | | | | 1 |
|  |  |  |  |  | Control | 0.71 | 99.4 | <0.01 | | 2.03 (1.77 to 2.34) |
| Weekend | 8038 | | 15.8% | 46 | 16/08/2019-19/08/2019 |  | | | | 1 |
|  |  |  |  |  | 26/04/2019-29/04/2019 | 1.02 | 13.0 | <0.01 | | 2.78 (1.59 to 4.84) |
|  |  |  |  |  | 13/12/2019-15/12/2019 | -1.20 | 9.0 | <0.01 | | 0.30 (0.14 to 0.66) |
| Time period | 8038 | | 15.8% | 2 | Day |  | | | | 1 |
|  |  |  |  |  | Evening | -0.17 | 5.5 | 0.02 | | 0.84 (0.73 to 0.97) |
|  |  |  |  |  | Night | 0.08 | 1.01 | 0.31 | | 1.09 (0.93 to 1.27) |
| Subjective crowding at the ED | 3279 | | 13.3% | 2 | Normal |  | | | | 1 |
|  |  |  |  |  | Quiet | 0.60 | 15.9 | <0.01 | | 1.82 (1.36 to 2.44) |
|  |  |  |  |  | Busy | 0.28 | 3.7 | 0.06 | | 1.32 (2.99 to 1.76) |
| **Nurse characteristics** | | | | | | | | | | |
| Nurse | 7635 | | 16.4% | 21 | Nurse 9 |  | | | | 1 |
|  |  |  |  |  | Nurse 7 | 0.89 | 24.3 | <0.01 | | 2.46 (1.72 to 3.52) |
|  |  |  |  |  | Nurse 19 | -0.73 | 7.4 | 0.01 | | 0.48 (0.28 to 0.81) |

DF: degrees of freedom
MTS: Manchester Triage System
ED: Emergency Department
ORL: Otorhinolaryngology
*: Only for urgency categories four and five because the primary outcome was zero in the other categories
