# Supplementary material for: Triaging and referring in adjacent general and emergency departments (the TRIAGE trial): A cluster randomised controlled trial
Source: PLoS One. 2021 Nov 3;16(11):e0258561. doi: 10.1371/journal.pone.0258561 (PMC8565772; doi:10.1371/journal.pone.0258561)
Supplement: S2 Table — ED: Emergency Department. GP: General Practitioner. MTS: Manchester Triage System. (DOCX) [file pone.0258561.s010.docx]

**S2 Table.** Characteristics of patients referred back to the ED after triage to the GPC

| Age | Sex | eMTS presentational flow chart | GP diagnosis | Hospital care | Admission |
| --- | --- | --- | --- | --- | --- |
| 17 | MALE | Falls | Concussion | Imaging | No |
| 18 | FEMALE | Back pain | Sciatica | None | No |
| 33 | FEMALE | Abdominal pain in adults | Localised abdominal pain | None | No |
| 13 | MALE | Limb problems | Musculoskeletal injury | None | No |
| 18 | FEMALE | Unwell adult | Unwell | Imaging | No |
| 9 | MALE | Headache | Headache | Imaging | No |
| 18 | FEMALE | Abdominal pain in adults | In labour | None | No |
| 87 | MALE | Wounds | Cut/laceration | Imaging | No |
| 47 | FEMALE | Unwell adult | Headache | Monitoring of vital signs | Yes |
| 76 | MALE | Abdominal pain in adults | Kidney stones | None | No |
| 35 | FEMALE | Abdominal pain in adults | Unspecified illness | Monitoring of vital signs | No |
| 11 | MALE | Headache | Symptoms of the nervous system | None | Yes |
| 18 | FEMALE | Abdominal pain in adults | Kidney stones | None | No |
| 22 | FEMALE | Limb problems | Musculoskeletal injury | None | No |
| 44 | FEMALE | Limb problems | Unspecified fracture | None | No |
| 21 | FEMALE | Headache | Headache | None | No |
| 27 | FEMALE | Limb problems | Musculoskeletal injury | None | No |
| 27 | FEMALE | Abdominal pain in adults | Generalised abdominal pain | None | No |
| 1 | MALE | Unwell baby | Fever | None | No |
| 9 | MALE | Abdominal pain in children | Unspecified illness | None | Yes |
| 53 | FEMALE | Headache | Unspecified illness | None | No |
| 32 | MALE | Abdominal pain in adults | Unspecified illness | None | No |
| 1 | FEMALE | Unwell child | Fever | Monitoring of vital signs | No |
| 26 | FEMALE | Back pain | Symptoms/complaints regarding back pain | None | No |

MTS: Manchester Triage System
GP: General Practitioner
