# Supplementary material for: Triaging and referring in adjacent general and emergency departments (the TRIAGE trial): A cluster randomised controlled trial
Source: PLoS One. 2021 Nov 3;16(11):e0258561. doi: 10.1371/journal.pone.0258561 (PMC8565772; doi:10.1371/journal.pone.0258561)
Supplement: S3 Table — MTS: Manchester Triage System. Df: degrees of freedom. (DOCX) [file pone.0258561.s011.docx]

**S3 Table.** Generalised Mixed Model for the primary outcome

| Determinant | Chi square | Df | P-value |
| --- | --- | --- | --- |
| MTS flow chart category | 567.3 | 15 | <0.01 |
| Admission type | 51.2 | 1 | <0.01 |
| Nurse identifier | 144.5 | 22 | <.0.01 |
| Subjective crowding | 15.4 | 3 | <0.01 |
| Residence | 11.5 | 2 | <0.01 |
| Socioeconomic status | 21.9 | 2 | <0.01 |
| Time period | 42.5 | 3 | <0.01 |
| Age | 12.2 | 5 | 0.032 |

MTS: Manchester Triage System

Df: degrees of freedom
