# Supplementary material for: Triaging and referring in adjacent general and emergency departments (the TRIAGE trial): A cluster randomised controlled trial
Source: PLoS One. 2021 Nov 3;16(11):e0258561. doi: 10.1371/journal.pone.0258561 (PMC8565772; doi:10.1371/journal.pone.0258561)
Supplement: S4 Table — ORL: Otorhinolaryngology. (DOCX) [file pone.0258561.s012.docx]

**S4 Table.** presentational flow chart categories

| Diarrhoea and vomiting | Abdominal complaints |
| --- | --- |
| Abdominal pain in adults | Abdominal complaints |
| Gastro-intestinal bleeding | Abdominal complaints |
| Neck Pain | Back and neck pain |
| Back Pain | Back and neck pain |
| Chest pain | Chest pain |
| Worried parent | Children |
| Abdominal pain in children | Children |
| Crying baby | Children |
| Shortness of breath in children | Children |
| Limping Child | Children |
| Abused or neglected child | Children |
| Unwell child | Children |
| Unwell baby | Children |
| Unwell newborn | Children |
| Eye problems | Eye problems |
| Limb Problems | Limb Problems |
| Apparently drunk | Mental complaints |
| Mental illness | Mental complaints |
| Behaving strangely | Mental complaints |
| Headache | Neurological complaints |
| Fits | Neurological complaints |
| Facial problems | ORL complaints |
| Dental problems | ORL complaints |
| Sore throat | ORL complaints |
| Ear problems | ORL complaints |
| Palpitations | Others |
| Abscesses and local infections | Others |
| Allergy | Others |
| Diabetes | Others |
| Rashes | Others |
| Asthma | Respiratory complaints |
| Shortness of breath in adults | Respiratory complaints |
| Bites and stings | Trauma and accidents |
| Chemical exposure | Trauma and accidents |
| Falls | Trauma and accidents |
| Head injury | Trauma and accidents |
| Overdose and poisoning | Trauma and accidents |
| Chest injury | Trauma and accidents |
| Assault | Trauma and accidents |
| Major incident | Trauma and accidents |
| Collapse | Unwell Adult |
| Unwell adult | Unwell Adult |
| Sexually acquired infection | Urinary or gynaecological complaints |
| Testicular pain | Urinary or gynaecological complaints |
| Urinary problems | Urinary or gynaecological complaints |
| Per Vaginam bleeding | Urinary or gynaecological complaints |
| Pregnancy | Urinary or gynaecological complaints |
| Burns and scalds | Wounds |
| Foreign body | Wounds |
| Wounds | Wounds |

ORL: Otorhinolaryngology
