# Supplementary material for: Triaging and referring in adjacent general and emergency departments (the TRIAGE trial): A cluster randomised controlled trial
Source: PLoS One. 2021 Nov 3;16(11):e0258561. doi: 10.1371/journal.pone.0258561 (PMC8565772; doi:10.1371/journal.pone.0258561)
Supplement: S2 File — Overview of the minor changes made to the study protocol after trial registration. (DOCX) [file pone.0258561.s014.docx]

**S2 File. Minor changes to the study protocol.** Overview of the minor changes made to the study protocol after trial registration.

## Outcome measures

- The description of the primary and secondary outcomes have been modified to make the report more readable without changing their meaning
- In the original study protocol (but not in the trial registration!) the objectives were described as hypothesis. The authors have purposely chosen to register and report the trial as objective driven in stead of hypothesis driven as these hypothesis were not clearly enough defined and not always in line with the used methodology
- The terminology was slightly changed: the first ‘other outcome’ measure was reformulated and because of its importance renamed as the ‘secondary outcome’. All remaining secondary and other outcomes were renamed as ‘additional outcomes’.
- Association between the primary outcome and presenting complaint expressed as the title of a Manchester Triage System presentation: reporting about 53 different categories is unreadable and raises statistical issues because some categories were seldomly chosen. We have re-categorised them into 15 clinically relevant categories. (see supplementary table 4)
- Association between the primary outcome and subjective workload at the ED as judged by the emergency nurse: the number of categories of this variable has been reduced from four to three because “uncontrollable busy” was rarely chosen.
- Association between the primary outcome and subjective workload at the GPC: this variable was ultimately not available to the researchers as they were only allowed to collect data from the included patients.
- Association between the primary outcome and the age of the patient: this variable was categorised into age intervals to make the report more readable/consistent
- Association between the primary outcome and the ZIP-code of the patient: to make this variable more relevant to an international audience, it was dichotomised into nearby (communities surrounding the ED and covered by the GPC) or not nearby (all other communities)
- Association between the primary outcome and the season: there was a correlation with the primary outcome: winter versus other seasons (OR 1.47, 95%CI 1.23 to 1.92) but the only clusters during winter were Christmas, new year’s evening and the first three weekends of the trial making this finding prone to bias. There was no difference among the other seasons. To simplify the report this variable has been left out.
- Association between the primary outcome and the hour of the day of the patient's presentation: the analysis of this variable with 24 categories was hard to interpret and has been replaced by a more clinically relevant division: day, evening and night.
- ED Physician’s opinion on the ideal allocation of the patient: these physician’s had four options: ED, both ED and GPC, GPC or delay of care. Because delay of care is currently not allowed in Belgium and because the authors wanted to report predictive values, this variable has been dichotomised.
- Number of patients who returned to the ED within two weeks (in the protocol, not in the registration): this variable was ultimately not available to the researchers
- Sensitivity and specificity: positive and negative likelihood ratios were reported instead, see the statistical analysis plan for details
- The primary outcome after the trial ended was added as an additional outcome.

## Eligibility Criteria

- Although always the intention, the exclusion of referred patients was not explicitly mentioned as an exclusion criterium (flaw in the study protocol).
