# Supplementary material for: Triaging and referring in adjacent general and emergency departments (the TRIAGE trial): A cluster randomised controlled trial
Source: PLoS One. 2021 Nov 3;16(11):e0258561. doi: 10.1371/journal.pone.0258561 (PMC8565772; doi:10.1371/journal.pone.0258561)
Supplement: Attachment — Submitted filename: TRIAGE trial PLOS ONE Rebuttal letter.docx [file pone.0258561.s016.docx]

# Dear editor, dear reviewers,

We were pleased to read that PLOS ONE wants to publish our work and appreciate the suggestions for revision. We believe we have addressed all of them and are willing to make additional changes if necessary. Please find our response to the raised issues in the table below.

| **Comment** | **Response** |
| --- | --- |
| Having intensively reviewed your revised draft… | We think there has been a misunderstanding about the history of our submission. The current version is our first revision, we did not submit any revision before, but the manuscript was returned to us because of a small administrative error. |
| Please ensure that your manuscript meets PLOS ONE's style requirements, including those for file naming | We have double checked these requirements and adjusted the paper accordingly |
| We note that the grant information you provided in the ‘Funding Information’ and ‘Financial Disclosure’ sections do not match.  When you resubmit, please ensure that you provide the correct grant numbers for the awards you received for your study in the ‘Funding Information’ section. | We now provide a correct financial disclosure statement in revised cover letter. We could, however, only add the name of the sponsor in editorial manager by selecting its Dutch name. That is why we have changed the English to the Dutch name in our financial disclosure statement. |
| Please upload your figure files to the Picture Analysis and Conversion Engine (PACE) digital diagnostic tool | All revised figures have now passed PACE successfully |
| Abstract Maximum word count is 300 | We have shortened the abstract to comply with this requirement |
| "(...) nurse-led triage using a new tool assigning patients (...)" - meaning remains unclear, please add information. | We have made this sentence clearer. |
| "(...) in the control group 431/1744 (24.7%, 95% CI 22.7 to 26.8)." Please provide complete sentences | We have re-written this phrase into a complete sentence |
| - What is meant when referring to "MTS presentational flowchart"? Please clarify. | We have changed this unclear technical term to the more understandable term “reason for encounter”. Because the term presentational flowchart is more concise, we have chosen to keep it in the body of the paper and explain its meaning in the methods section. |
| - "ED nurses using a new tool safely diverted 9.5% of the included patients to primary care." This does not refer to your aims/objectives. Please revise carefully. | The conclusion did indeed not match the objectives. We have updated the objectives which are now in line with the conclusions. |
| . Revise referencing, "(...) in a primary care setting.[1-4]" must read "(...) in a primary care setting [1-4]." Revise thoroughly. | Alle references have been re-located before the full stop. |
| - "Three small non randomised trials about the MTS and diversion to primary care revealed promising results [14-16]." Rationale would seem unclear. Please elaborate both aims and objectives more clearly. | We have rewritten our objectives and improved the paragraph about study rationale. |
| - What about your null hypothesis? Remember that H0 must be deducible from the foregoing thoughts. | We have rewritten the study objectives and added our previously implicit null hypothesis to the analysis section. |
| Revise for "Material and methods". | We have revised the title of this section |
| Please stick to H0 when starting the discussion | We have explicitly added the rejection of the null hypothesis to the beginning of the discussion section. |
| - "The referral rate of patients in the primary outcome to the ED (4%) was similar to the referral rate of the studied GPC for untriaged patients (6%) and in general practice OOH services in the United Kingdom (8.1%)." Reference missing. | We have fixed this missing citation. |
| - With your Conclusions, please stick exclusively to your revised aims. Do not simply repeat your results here. Instead, provide a reasonable and generalizable extension of your outcome. | The conclusions are now in line with our revised objectives/aim. We have added an extra sentence about the impact of our findings. |
| Refs - Please revise for uniform formatting. Compare "The American journal of emergency medicine. 2011;29(3):333-45.", "BMJ. 2020;368:m462. doi:10.1136/bmj.m462.", or "Journal of emergency management (Weston, Mass). 2016;14(5):349-64. doi: 10.5055/jem.2016.0300. PubMed PMID: 27873299.", and re-edit thoroughly. | We have re-edited all references |
| doi and PMID numbers will be sufficient. | We have provided a DOI for each reference as an alternative to traditional volume and page numbers. |
| In the methods section it would be interesting to indicate which diagrams were not changed in this new eMTS version. | We have added the titles of these flowcharts to the material section |
| In the results section it is indicated that the majority of patients assigned to GPC were a blue code, it would be interesting to compare the proportions (total blue patients and percentage of patients who used GPC vs total green patients and percentage of patients who used GPC). This is because in the most well-known MTS studies it is known that the majority of patients are green and a small proportion blue, this could give an accurate idea of the impact of this strategy. | The number of included patients in urgency category four (blue) can be found in table 1 and is indeed low. We have added the proportion of the primary outcome for urgency categories four and five to the results section.  The majority of the patients assigned to the GPC were green, not blue. We think the reviewer was confused by our unclear use of the word “more” in the sentence “CHAID analysis of the study tool parameters (S1 Supplementary Figure 1) showed that the urgency category was primordial: none of the patients in the three highest urgency categories was seen at the GPC, and **more** patients from urgency category five were diverted to the GPC as compared to urgency category four.” We have corrected this sentence. |
| Also, in the results section it is indicated that most of the patients eligible for GPC were detected on weekends, why? Are there more weekend accesses, are there no other types of services presents? Are the proportions of codes equal between weekend and week (or are there more green and blue due to the absence of some services)? | The GPC was only open during the weekends, so we conducted this trial only during weekends (and bank holidays, reported together in the paper as “weekends”). Unfortunately, we do not have data about the week to answer these important questions, a point we have added to the limitations of this study. |
| On page 17 it is stated that one patient elected for the GPC strategy died. Regardless of the individual case, this may also be the case in other non-specific symptom presentations that result in a more serious underlying condition (epigastric pain in a diabetic patient that results in a acute myocardial infarction, mild abdominal pain that results in appendicitis). There are different reports on individual symptom presentations and the functionality of MTS, and some of these indicate that patients with green and blue codes have a subsequent severe and time-dependent pathology (DOI: 10.1016/j.ienj.2020.100842; DOI: 10.1177/1474515118777402; DOI: 10.1111/jocn.15635). This might be an interesting aspect to be addressed in the discussion. | We want to thank the reviewer for this suggestion. We have added two phrases regarding commonly missed diagnosis to the paragraph in the discussion focussing on safety and we have added a new paragraph discussing the consequence of the patient that died. |
| In supplementary table S9-T2 it is reported that a patient with adult unwell chart presented with the diagnosis of headache and so on, was the correct application of MTS assessed? If not, it could result as a limitation of the study. | We did indeed not assess the correct application of the MTS, so we have added this aspect to the limitations of our study. The diagnoses as listed in table S9-T2 are those registered by the GP after their consultation. The GPs were only allowed to select one diagnosis out of a list, so it is not possible to assess the correctness of the triage based on this diagnosis alone. |
| Another aspect that could be included as a limitation of the study is the fact that the GPC physician may have altered their performance in patients sent from triage. Could he have decreased the rates of patients sent back to the ED given this knowledge? This limitation is given by the type of study but still deserves to be mentioned in the limitations section. | We have added this point to our discussion. |
| Table 1: State the statistical testing methods used to estimate the p-values. | We have added the requested information |
| 2- Table 2: To improve clarity, modify the title to include “logistic regression bivariate analysis.” | We have changed the title of this table accordingly. |

Sincerely,

The Authors
